# Medical education and leadership: a call to action for Brazil’s mental health system

**DOI:** 10.5116/ijme.5b1b.b0a2

**Published:** 2018-06-22

**Authors:** Igor D. Bandeira, John Mendoza

**Affiliations:** 1 Brain and Mind Centre, The University of Sydney, Sydney, NSW, Australia

**Keywords:** Medical education, leadership, call to action, Brazil, mental health system

## Introduction

Brazil is the largest country in South America, with approximately 206 million inhabitants. The World Bank recognises Brazil as an upper middle-income nation due to the size of its economy however, despite its economic wealth, there is a high degree of social inequality due to uneven economic distribution.^1^

Health is considered a basic right in Brazil, which the Government is required to administer and provide through a universal public system. The Unified Health System (Sistema Único de Saúde - SUS) is administered by federal, state and municipal governments and its core principles include decentralisation of care, universality of access and equality of rights. Brazil’s Mental Health Policy is part of the Unified Health System and guarantees the civil rights of patients suffering from mental illnesses, while promoting integrated care in community centres for psychiatric assistance rather than hospitalisation, which is generally reserved for patients with severe conditions.^2^

The order in which assistance for mental health patients in Brazil is prescribed is based on the severity of symptoms and on the illness itself. Mild and moderate disorders (e.g., depression and anxiety) are most commonly treated in a primary care facility by family doctors. More severe disorders, such as schizophrenia and substance abuse, are treated at Psychosocial Care Centres (Centro de Atenção Psicossocial - CAPS), by psychiatrists and psychologists.^3^^,^^4^ Although, in theory, this may seem an ideal model for mental health assistance, it does not always work in practice. A significant limitation includes a general lack of professionally qualified family doctors, who are often unable to diagnose or treat even mild disorders.^5^^,^^6^ Since there are few specialised psychiatric clinics operating within the Unified Health System, a large proportion of mental health patients are unable to receive adequate treatment.^7^

Additionally, another limitation is physical access to the health system. The distribution of specialised and general clinics is extremely uneven across different regions of Brazil, making it impossible to implement a Mental Health Policy with the same unifying standards for the entire country.^4^^,^^7^^,^^8^ Underinvestment in Brazil’s healthcare system not only contributes to an overall lack of clinics but also to a spread of under-resourced clinics across the country. This lack of adequate funding not only inhibits the creation of outpatient clinics but also the necessary expansion of professional training and networks, inevitably leading to under-diagnosis, inefficient treatment and poor patient management.^4^^,^^7^^-^^10^

A similar mental health profile can also be found in other low to middle-income countries, making these problems a global concern.^11^^-^^13^ The lack of quality training in public health, the resistance of psychiatrists to promote non-psychiatrists into senior management positions and the volume of complex clinical and hospital management tasks designated to leaders are just some of the major obstacles in improving mental health systems in many developing countries.^13^

### A Way Forward

The purpose of this article is to analyse how leadership models and constructs can be implemented within management strategies to identify, address and alleviate the mental health assistance issues highlighted. The lack of management and leadership training among community leaders has been identified as one of the major barriers to enhancing the quality of mental health services in low to middle-income countries.^13^^,^^14^ Therefore, creating new management strategies based on leadership could generate creative solutions for recurring issues and problems within the Brazilian mental health system.

Leadership has many definitions and concepts.^15^ According to Scherr and Jensen, leadership can be defined as a set of verbal and nonverbal actions that may lead to results that otherwise could not be achieved.^16^ In this article we will adopt two fundamental theories: transformational and transactional leadership. Transformational leadership involves behaviours based on interpersonal relationships^17^ that can be explained by some key concepts: inspirational motivation (construction of a behaviour that inspires and motivates other group members); individual consideration (the leader supports individual development); idealized influence (leader’s actions are carried out in a consistent and ethical manner); and intellectual stimulation (reinforcement of creative and innovative thinking).^18^ On the other hand, the concept of transactional leadership involves realization of domain-specific tasks, centred around past events.^17^

In general, doctors and health professionals have a variety of responsibilities which often require inherent leadership qualities due to the specialisation and expertise of their role. The need to develop leadership skills is recognised as one of the most important goals for medical education this century^19^^-^^21^ however, the inclusion of this subject in the medical curriculum is not compulsory or standardized across countries.^20^^,^^22^^,^^23^ In Brazil, according to the national curriculum guidelines for medical education, leadership skills are part of the basic knowledge that a general doctor must have in order to work in multidisciplinary teams with responsibility, empathy, effective communication and proper planning for decision making, as well as being able to assume leadership positions.^24^ However, in spite of this, most medical schools do not integrate leadership skills into the curriculum, which creates a significant gap between what is expected in terms of a basic skill set of a doctor and the actual profile of a recently graduated medical professional.

Moreover, there are currently no studies reporting or analysing leadership education in Brazil or as to whether the process of including leadership skills in medical practices has been effective. Much of the efforts employed in leadership training are based on responsibility for extracurricular activities. Thus, leadership and management skills can be acquired indirectly through this parallel curriculum created by students and professors within institutions to fill gaps in teaching but not by the formal inclusion of leadership training within academic institutions.^25^^,^^26^

There are many reasons why encouraging leadership is essential to the improvement of Brazil’s mental health system. The process of identifying and training leaders can make an important contribution to realizing the Unified Health System's principles while improving the quality of mental health policies and assistance throughout the country.^27^ This process requires a shared vision between the various agents involved to make the necessary changes to improve the quality of services. Trained leaders will have a crucial impact, not only in inspiring other staff members but also on the broader community, motivating individuals to remain committed to the core principles of the Unified Health System.^28^

Partnerships between health professionals, individuals affected by mental illness and caregivers can be essential to the process of leadership building due to the experience and knowledge that are required for the development of public policies, their evaluation, and the enhancement of quality of services.^29^ This relationship between leaders from various sectors can even assist the development of research and educational interventions in the community.^28^

Leadership training needs to be distributed among health professionals and users of the health system, strengthening interpersonal relationships as well as team dynamics.^30^ This can facilitate communication among the different sectors involved in primary health care and mental health services, as well as engage the community in demanding improvements in quality and access to health care.

In addition, the interaction between the different Brazilian medical societies is critical in improving and modifying the medical school curriculum. Such action may facilitate the creation of new leaders in the future, giving longevity to the process of improvement in mental health services.

## Conclusions

In summary, it is necessary to encourage the learning and teaching of leadership skills in medical schools to improve healthcare outcomes in Brazil. Moreover, healthcare providers who can sustainably manage resources, solve problems and make informed decisions are needed to guarantee the improvement of Brazil’s Mental Health System. By learning leadership skills, it is possible to create an environment that stimulates research, as well as provide practical and creative solutions to several previously reported issues. In this way, the empowering of new leaders can have a significant impact on the quality of health services, particularly in the light of the proposed decentralized mental health assistance in Brazil.

### Acknowledgements

We acknowledge the National Council for Scientific and Technological Development (Conselho Nacional de Desenvolvimento Científico e Tecnológico - CNPq, Brazil) for funding the scholarship of the primary author.

### Conflicts of Interest

The authors declare that they have no conflict of interest.
